# Ophthalmic In Situ Gelling System Containing Lanosterol Nanoparticles Delays Collapse of Lens Structure in Shumiya Cataract Rats

**DOI:** 10.3390/pharmaceutics12070629

**Published:** 2020-07-04

**Authors:** Noriaki Nagai, Kazuki Umachi, Hiroko Otake, Mikako Oka, Noriko Hiramatsu, Hiroshi Sasaki, Naoki Yamamoto

**Affiliations:** 1 Faculty of Pharmacy, Kindai University, 3-4-1 Kowakae, Higashi-Osaka, Osaka 577-8502, Japan; 1611610075d@kindai.ac.jp (K.U.); hotake@phar.kindai.ac.jp (H.O.); 2 Laboratory of Clinical Pharmacology, Yokohama University of Pharmacy, Yokohama, Kanagawa 245-0066, Japan; m.oka@hamayaku.ac.jp; 3 Laboratory of Molecularbiology and Histochemistry, Fujita Health University Institute of Joint Research, 1-98 Dengakugakubo, Kutsukake, Toyoake 470-1192, Aichi, Japan; norikoh@fujita-hu.ac.jp; 4 Department of Ophthalmology, Kanazawa Medical University, 1-1 Daigaku, Uchinada, Kahoku, Ishikawa 920-0293, Japan; sasaki-h@k5.dion.ne.jp (H.S.); naokiy@kanazawa-med.ac.jp (N.Y.)

**Keywords:** nanoparticles, lanosterol, cataract, in situ gel, Shumiya cataract rat

## Abstract

We attempted to prepare ophthalmic in situ gel formulations containing lanosterol (Lan) nanoparticles (LA-NPs/ISG) and investigated the characteristics, delivery pathway into the lens, and anti-cataract effects of LA-NPs/ISG using SCR-N (rats with slight lens structure collapse) and SCR-C (rats with a combination of remarkable lens structure collapse and opacification). LA-NPs/ISG was prepared by bead milling of the dispersions containing 0.5% Lan powder, 5% 2-hydroxypropyl-β-cyclodextrin, 0.5% methylcellulose, 0.005% benzalkonium chloride, and 0.5% mannitol. The particle size distribution of Lan was 60–250 nm. The LA-NPs/ISG was gelled at 37 °C, and the LA-NPs/ISG was taken into the cornea by energy-dependent endocytosis and then released to the intraocular side. In addition, the Lan contents in the lenses of both SCR-N and SCR-C were increased by the repetitive instillation of LA-NPs/ISG (twice per day). The space and structure collapse in the lens of SCR-N with aging was attenuated by the instillation of LA-NPs/ISG. Moreover, the repetitive instillation of LA-NPs/ISG attenuated the changes in cataract-related factors (the enhancement of nitric oxide levels, calpain activity, lipid peroxidation levels, Ca^2+^ contents, and the decrease of Ca^2+^-ATPase activity) in the lenses of SCR-C, and the repetitive instillation of LA-NPs/ISG delayed the onset of opacification in the SCR-C. It is possible that the LA-NPs/ISG is useful in maintaining lens homeostasis.

## 1. Introduction

A cataract is a form of ophthalmic disease involving the collapse of tissue structure via crystallin aggregation and lens opacification by excessive collapse, and it is the leading global cause of human blindness. In addition, even slight collapses of tissue structure can have impacts such as sleep disturbance [1], disorientation [2], and cognitive impairment [3], and they can contribute to falls in the elderly [4]. Many risk factors for the onset of cataracts have been reported, including exposure to UV light, oxidative stress, genetic predisposition, aging, toxic agents, inherited mutations, metabolic disorders, and diabetes. In clinical settings, the effective prevention strategy for cataracts is surgery, and no anti-cataract drugs are available thus far. Nonetheless, researchers recently demonstrated that lanosterol (Lan), which is a key early rate-limiting step in the biosynthesis of cholesterol, disrupted the aggregation of γD-crystalline by binding to the hydrophobic dimerization interface in humans [5], and it played a key role in the prevention of cataract formation in animal models [6].

Eye drops are accepted by many patients because of their safety and simplicity, and they are considered the preferred route for therapy of ophthalmic diseases. However, traditional ophthalmic formulations cannot provide an adequate drug concentration in lenses, and only 5% of the instilled drug can penetrate the cornea. Thus, the low bioavailability of eye drops is a problem in cataract therapy. The barriers of the tear film and cornea are related to topical ocular drug delivery [7], and it is important to design a drug delivery system to improve bioavailability.

It is generally considered that the prolongation of preocular residence and enhancement of corneal permeability overcomes the low ocular drug bioavailability [8]. Several attempts, such as in situ gels, ointments, ion-triggered release [9], preformed gels [10], bio-adhesive polymers [11], and drug-loaded contact lens triggered by pH [12] have been made to improve drug residence time. Moreover, various drug delivery systems, such as microparticles, nanoparticles, nanostructured lipid carriers, nanosuspensions, nanocrystals, liposomes, and dendrimers have been studied to overcome issues relating to the absorption, retention, and stability of drugs [13,14,15]. In previous research, we also designed disulfiram and indomethacin nanoparticles using the bead mill, and showed that the adsorption to the surface of cyclodextrin decreases the cohesion of nanoparticulate solids, and the addition of 2-hydroxypropyl-β-cyclodextrin (HPCD) was suitable for the preparation of nanoparticles using mill methods [16,17]. Furthermore, we found that energy-dependent endocytosis was related to the transcorneal penetration of ophthalmic formulations containing nanoparticles [16,18], and the instillation of ophthalmic formulations containing nanoparticles can deliver the drug into the lens [18]. In addition, in order to further improve contact with the eye surface, we prepared an in situ gelling system based on methylcellulose (MC) and solid nanoparticles, and showed that the in situ gel containing nanoparticles provided high absorption, retention, and stability of drugs [17]. This combination of nanoparticles and an in situ gelling system may be useful for the development of ophthalmic formulations for anti-cataract treatments.

In the development of anti-cataract ophthalmic formulations, animal models have greatly contributed. The Shumiya cataract rat (SCR) is a hereditary cataractous rat strain, which is classed as two models (non-cataract type, SCR-N, and cataractous type, SCR-C) [19]. SCR-N (ctr1 × ctr1, Ctr2 × Ctr2), without the mutations causing premature cataracts, maintains a transparent lens, although slight lens structure collapse was observed at 8–12 weeks of age [20]. In contrast, SCR-C (ctr1 × ctr1, Ctr2 × Ctr2^l^) carries a specific combination of hypomorphic mutations of the genes of FDFT1 (farnesyl diphosphate farnesyl transferase 1) and Lan synthase, and opacification with significant lens structure collapse was seen at 9–10 weeks of age [20]. In addition, calpain activation via the increase in calcium content in the lens and the enhancement of inducible nitric oxide (NO) synthase (iNOS) were reported during lens opacification [21,22]. Thus, the SCR is a useful model in studies to evaluate Lan nanoparticles in in situ gel for ophthalmic delivery. In this study, we attempted to prepare an ophthalmic in situ gel formulation containing Lan nanoparticles (LA-NPs/ISG) and investigated the characteristics, delivery pathway into the lens, and anti-cataract effects of LA-NPs/ISG using rats with slight lens structure collapse (SCR-N) and rats with a combination of remarkable lens structure collapse and opacification (SCR-C).

## 2. Materials and Methods

### 2.1. Chemicals

Lan, rottlerin, dynasore, and Cell Count Reagent SF were purchased from Nacalai Tesque, Inc. (Kyoto, Japan), and a lipid peroxidation (LPO) assay kit (BIOXYTECH^®^ LPO-586^TM^) was provided by OXIS International, Inc. (Portland, Oregon, USA). Nystatin was obtained from Sigma-Aldrich Japan (Tokyo, Japan). Heat-inactivated fetal bovine serum, penicillin, streptomycin, and Dulbecco’s modified Eagle’s medium/Ham’s F12 (DMEM/F12) were purchased from GIBCO (Tokyo, Japan). Benzalkonium chloride (BAC) and a Bio-Rad protein assay kit were obtained from Kanto Chemical Co., Inc. (Tokyo, Japan) and Bio-Rad Laboratories (Hercules, CA, USA), respectively. Ca test kits, mannitol, cytochalasin D, and isoflurane were obtained from Wako Pure Chemical Industries, Ltd. (Osaka, Japan), and 0.1% pivalephrine and 0.4% benoxil were purchased from Santen Pharmaceutical Co., Ltd. (Osaka, Japan). MC (type SM-4) and HPCD was supplied by Shin-Etsu Chemical Co., Ltd. (Tokyo, Japan) and Nihon Shokuhin Kako Co., Ltd. (Tokyo, Japan), respectively. SUPER FIX was provided by Kurabo Industries (Osaka, Japan). Calpain Activity Fluorometric Assay Kits were purchased from BioVision Inc. (San Francisco, CA, USA).

### 2.2. Animals

Male SCR aged 6–12 weeks with (SCR-C) or without (SCR-N) lens opacification were selected to evaluate the therapeutic potential for anti-cataract treatment, and Japanese albino rabbits (approximately 2.7 kg) were used to investigate the corneal toxicity and transcorneal penetration of ophthalmic formulations. These rats and rabbits were housed under normal conditions. The experiments using the animals were approved by the Committee for Animal Experiments at Kindai University on 1 April 2013, and the project identification code was KAPS-25-003. The instillation was performed under isoflurane anesthesia, and 30 microliters of LA-NPs/ISG containing 0.5% Lan were repetitively instilled twice per day from 6 weeks of age for 6 weeks. All experiments were performed in accordance with the guidelines for the ARVO (Association for Research in Vision and Ophthalmology).

### 2.3. Preparation of LA-NPs/ISG

LA-NPs/ISG were prepared according to our previous reports [17,23,24]. The Lan powder (0.5%) was dispersed in purified water containing 5% HPCD, 0.5% MC, 0.005% BAC, and 0.5% mannitol, and milled with 0.1-mm zirconia beads by a Bead Smash 12 (Wakenyaku Co. Ltd., Kyoto, Japan) at 5500 rpm for 30 s at 4 °C. The mill treatment was repeated 20 times. Then, saline containing 5% HPCD, 0.5% MC, 0.005% BAC, and 0.5% mannitol, and the dispersions, were gently stirred to eliminate air bubbles generated by the bead mill. After the disappearance of air bubbles, the mill process using the Bead Smash 12 was carried out as before (5500 rpm, 30 s × 20 times, 4 °C). The milled dispersions containing 0.5% Lan, 0.005% BAC, 0.5% mannitol, 5% HPCD, and 1.5% MC were used as LA-NPs/ISG, and the pH was adjusted to 7. The Lan concentration was measured by a simple Liquid Chromatography (LA)-Charged Aerosol Detector (CAD) method. Twenty microliters of samples in methanol were injected, and the Lan in samples was separated by an LC-10AD pump (Shimadzu, Kyoto, Japan) with TSK gel ODS-100S (5 µm, 150 × 2.0 mm I.D., Tosoh Co., Tokyo, Japan), a step gradient experiment was performed using 5% and 100% methanol over 19 min (flow rate of 0.4 mL/min), and the Lan was detected by a Corona Veo detector (Thermo Fisher Scientific, Inc., Waltham, MA, USA). The charger voltage, charger current, and inlet pressure (nitrogen) were 2.66 kV, 0.99 µA, and 61.9 psi, respectively [24].

### 2.4. Measurement of Characteristics of LA-NPs/ISG

A SALD-7100 (Shimadzu Corp., Kyoto, Japan) and NANOSIGHT LM10 (QuantumDesign Japan, Tokyo, Japan) were used to measure the size of Lan particles, and the refractive index in SALD-7100 was set to 1.60–0.10i. An SPM-9700 (Shimadzu Corp., Kyoto, Japan) was used to obtain the atomic force microscope (AFM) images. The LA-NPs/ISG were stored for 1 month at 20 °C after preparation, the stored LA-NPs/ISG were collected from 5 mm under the surface over time, and the changes in Lan concentration in the collected samples represented dispersibility. An MCR302 attached to a CP50-1 was used to measure the viscosity of LA-NPs/ISG at 20 °C and 37 °C (Anton Paar Japan K.K, Tokyo, Japan). The shear rate and measurement time were 90–100 rpm/s and 2 s, respectively. The solubilized and non-solubilized Lan were separated by centrifugation at 100,000× *g* using an Optima^TM^ MAX-XP Ultracentrifuge (Beckman coulter, Osaka, Japan), and the levels of solubilized Lan were measured by the LC-CAD method described above. A Zeta Potential Meter Model 502 (Nihon Rufuto Co., Ltd., Tokyo, Japan) was used to measure the zeta potential of LA-NPs/ISG.

### 2.5. Evaluation of Cell Toxicity of LA-NPs/ISG Using Culture Human Corneal Epithelial Cell (HCE-T Cell)

Five percent (%, *v*/*v*) heat-inactivated fetal bovine serum, 1000 IU/mL penicillin, and 0.1 mg/mL streptomycin were added to DMEM/F12, and the HCE-T cells were cultured in this medium. A quantity of 1 × 10^4^ cells of HCE-T was seeded in 96-well microplates (IWAKI, Chiba, Japan) and incubated for 24 h. Saline, vehicle, and LA-NPs/ISG were added to the cell cultures, and the cells were stimulated for 120 s [23]. Then, the cells were washed by phosphate buffer and incubated in DMEM/F12 for 1 h. Following incubation, Cell Count Reagent SF was added and incubated for 1 h, and the absorbance (Abs) at 490 nm was measured. The cell viability was analyzed by Equation (1):

Cell viability (%) = Abs_treatment_/Abs_non-treatment_ × 100. (1)

### 2.6. Evaluation of Corneal Toxicity of LA-NPs/ISG Using Rabbits

LA-NPs/ISG (30 µL) were repetitively instilled in rabbits twice per day (09:00 and 19:00) for 1 month. Then, 30 µL of 1% fluorescein was instilled to stain the wound area, and the wound area was monitored by a TRC-50X (Topcon, Tokyo, Japan).

### 2.7. Evaluation of Corneal Toxicity of LA-NPs/ISG Using Rat Debrided Corneal Epithelium

SCR-N aged 12 weeks were anesthetized with 0.4% Benoxil and isoflurane, and the corneal epithelium was debrided. Thirty microliters of LA-NPs/ISG were instilled 3 times per day (09:00, 15:00, and 21:00), and the eye was observed by the TRC-50X. When monitoring with the TRC-50X, 30 µL of 1% fluorescein was instilled to stain the corneal wounds. The debrided areas in SCR-N instilled with saline and LA-NPs/ISG were 12.01 ± 0.44 mm^2^ (n = 5) and 12.09 ± 0.42 mm^2^ (n = 7), respectively.

### 2.8. Transcorneal Penetration of LA-NPs/ISG Using Isolated Rabbit Cornea

Rabbits were euthanized by injecting a lethal dose of pentobarbital into the marginal ear vein, and the corneas were collected and set on a methacrylate cell (transcorneal cell). The donor and reservoir chamber were filled with LA-NPs/ISG and 10 mM 4-(2-hydroxyethyl)-1-piperazineethanesulfonic acid (HEPES) buffer (pH 7.4) consisting of 1 mM K_2_HPO_4_, 5.5 mM glucose, 136.2 mM NaCl, 1.7 mM CaCl_2_, and 5.3 mM KCl. The experiments of transcorneal penetration were performed at 4 °C and 37 °C for 6 h, and the solution (sample) in the reservoir chamber was collected over time. The Lan concentration in collected samples was measured by the LC-CAD method described above.

### 2.9. Treatment of Inhibitor of Energy-Dependent Endocytosis in the Isolated Rabbit Cornea

In the experiments for transcorneal penetration using the methacrylate cell (transcorneal cell), the isolated rabbit cornea was set as described above. Caveolae-mediated endocytosis (CavME), clathrin-mediated endocytosis (CME), macropinocytosis (MP), and phagocytosis were inhibited by the treatment of 54 µM nystatin [25], 40 µM dynasore [26], 2 µM rottlerin [27], and 10 µM cytochalasin [25], respectively, and these inhibitors were dissolved in 0.5% DMSO and added to a reservoir chamber with HEPES buffer. The experiments of transcorneal penetration were performed at 37 °C for 6 h, and the solution (sample) in the reservoir chamber was collected over time. The Lan concentration in collected samples was measured by the LC-CAD method described above. The area under the drug–concentration–time curve in the reservoir chamber (*AUC*_0–6h_) was determined according to the trapezoidal rule up to 6 h.

### 2.10. Measurement of Lan Content in Rat Lenses

The SCRs were euthanized under deep isoflurane anesthesia, and the lenses were carefully collected. Four hundred microliters of methanol were added to the collected lenses and homogenized on ice. The homogenates were centrifuged at 20,400× *g* for 15 min at 4 °C, and the supernatants were used as samples for measurement. The Lan contents were measured by the LC-CAD method described above.

### 2.11. Evaluation of Lens Structure in the SCR-N Using Hematoxylin and Eosin (H.E.) Staining

The SCR-N were euthanized by injecting a lethal dose of pentobarbital, and the eyes were removed and fixed at room temperature for 2 days using SUPER FIX. Three micrometer paraffin serial sections were prepared by microtome, the fixed lenses were prepared in paraffin blocks, and H.E. staining was performed for morphological observation. A biological upright microscope (Power BX-51, Olympus, Tokyo, Japan) was used to observe the specimens.

### 2.12. Scheimpflug Slit Images in the SCR-C

Lenses of the SCR-C without anesthesia were dilated by 0.1% pivalephrine, and Scheimpflug slit images were monitored by an EAS-1000 (Nidek, Aichi, Japan). The transparent area in the lenses was analyzed by software connected with the EAS-1000 and expressed as pixels. The flash level, thread level, and slit length were set to 100, 100, and 4.2 mm, respectively [28].

### 2.13. Measurement of Cataract-Related Factors

The SCR were euthanized by injecting a lethal dose of pentobarbital, and the lenses were removed and homogenized in saline on ice. The homogenates were centrifuged at 20,400× *g* for 15 min at 4 °C, and the supernatants were used as samples. A flow-through spectrophotometer (NOD-10, Eicom, Kyoto, Japan) was used to measure the nitric oxide (NO) levels [29], which were expressed as the level of the NO_2_^−^ metabolite. The calpain activity was measured at 505 nm by a Calpain Activity Fluorometric Assay Kit according to the manufacturer’s instructions and represented as the ratio of calpain activity levels in SCR aged 6 weeks. The LPO levels were analyzed by measuring the lipid peroxidation products 4-hydroxynonenal and malondialdehyde using an LPO Assay Kit [30], and the Ca^2+^-ATPase activity was calculated as the difference in the Pi liberated from ATP measured in the presence and absence of Ca^2+^ [30]. A Ca Test Kit was used to measure the Ca^2+^ content in the lenses [31]. The protein levels were determined using a Bio-Rad Protein Assay Kit, and the protein was used to evaluate the LPO levels and Ca^2+^-ATPase activity.

### 2.14. Statistical Analysis

Statistical significance was determined by the Student’s *t*-test and ANOVA followed by Dunnett’s multiple comparison (*p* < 0.05), and data are expressed as the mean ± standard error (S.E.) of the mean.

## 3. Results

### 3.1. Corneal Toxicity in the Instillation of LA-NPs/ISG

**Figure 1:**
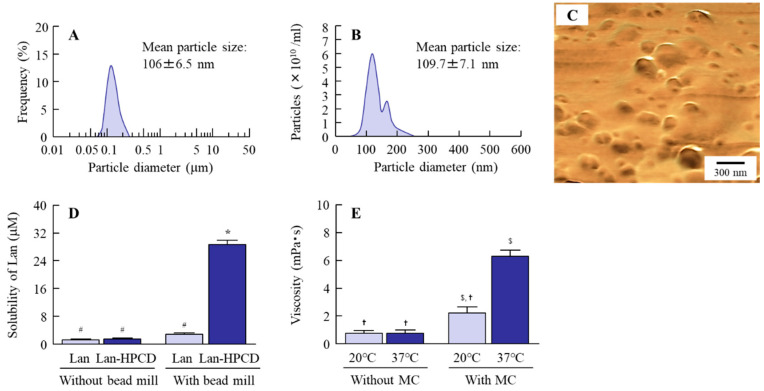
Evaluation of drug particle size, solubility, and viscosity in ophthalmic in situ gel formulations containing lanosterol (LA-NPs/ISG). (**A**,**B**) Particle size distribution of lanosterol (Lan) in LA-NPs/ISG using the SALD-7100 (**A**) and NANOSIGHT LM10 (**B**). (**C**) Atomic force microscope (AFM) image of Lan in LA-NPs/ISG. (**D**) Solubility of Lan in LA-NPs/ISG with or without 2-hydroxypropyl-β-cyclodextrin (HPCD). (**E**) Viscosity of ophthalmic Lan formulations with or without methylcellulose (MC) at 20 °C and 37 °C. n = 12. * *p* < 0.05, vs. Lan without bead mill. ^#^ *p* < 0.05, vs. Lan-HPCD with bead mill. ^$^ *p* < 0.05 vs. 20 °C without MC. ^✝^ *p* < 0.05 vs. 37 °C with MC. The bead mill treatment decreased the particle size of Lan to nano-size and increased the Lan/HPCD inclusion. The ratio of undissolved drug Lan was 99.8% in the 0.5% LA-NPs/ISG. The viscosity of LA-NPs/ISG was enhanced under the 37 °C condition.

**Figure 2:**
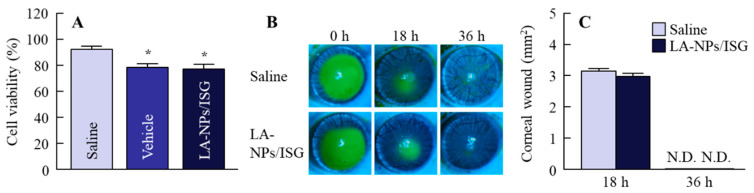
Corneal toxicity of LA-NPs/ISG in the HCE-T cells and rats. (**A**) Effect of LA-NPs/ISG on the viability in HCE-T cells. The HCE-T cells in 96-well microplates were treated with LA-NPs/ISG for 120 s. (**B**,**C**) Corneal images (**B**) and corneal wound healing (**C**) of rats repetitively instilled with LA-NPs/ISG. N.D., not detectable. Vehicle is solution consisting of benzalkonium chloride (BAC), mannitol, HPCD, and MC. n = 5–7. * *p* < 0.05, vs. Saline for each group. The viability in HCE-T cells treated with LA-NPs/ISG was similar to that of the vehicle, and the corneal wound healing showed no difference between that in the rats instilled with saline and LA-NPs/ISG.

First, we verified whether the Lan nanoparticles were able to be prepared, and we evaluated their solubility and viscosity at near body temperature (37 °C). The particle size distribution of Lan was decreased by the bead mill treatment and additives to a particle size of 60–250 nm (Figure 1A–C). In addition, the inclusion of Lan and HPCD (Lan/HPCD) was also increased by bead mill treatment, and the solubility of LA-NPs/ISG was 10.2-fold higher than that without HPCD (Figure 1D). The ratio of undissolved drug Lan was 99.8% in the 0.5% LA-NPs/ISG. The viscosity of Lan nanoformulations was enhanced by the addition of MC, the LA-NPs/ISG were gelled at 37 °C, and the viscosity of LA-NPs/ISG was 2.85-fold higher than that at 20 °C (Figure 1E). The zeta potential was low, with a level of −3.13 mV, although the Lan in LA-NPs/ISG was not aggregated for two weeks. The enhanced viscosity due to the addition of MC may have increased the stability of LA-NPs/ISG. In Figure 2, we show the corneal toxicity when the HCE-T cells and corneas of rats and rabbits were treated with LA-NPs/ISG. The treatment by vehicles consisting of BAC, mannitol, HPCD, and MC decreased the cell viability, which was approximately 85% of the saline-treated group. In other respects, Lan nanoparticles did not exhibit signs of cell toxicity, since cell viability was the same in HCE-T cells treated with the vehicle and with LA-NPs/ISG (Figure 2A). Moreover, it was examined whether the cornea of rabbits were damaged by the repetitive instillation of LA-NPs/ISG for one month (twice per day). No corneal injury due to instillation was observed in the rabbits. Furthermore, we demonstrated the effect of LA-NPs/ISG on corneal wound healing using rats’ debrided corneal epithelium (Figure 2B,C). The levels of corneal wound healing of rats instilled with saline were 3.12 ± 0.13 mm^2^ at 18 h, and wounds were cured 36 h after corneal epithelial abrasion. The corneal wound healing in the rats instilled with LA-NPs/ISG was similar to the rats instilled with saline.

### 3.2. Mechanism for Drug Delivery into Lens by the Instillation of LA-NPs/ISG

**Figure 3:**
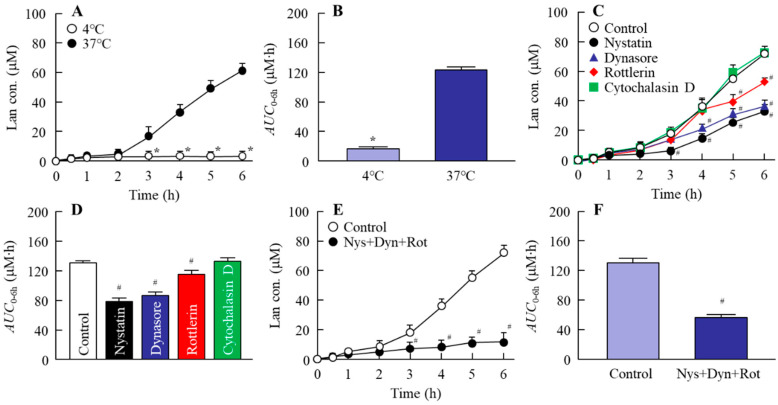
Relationships of energy-dependent endocytosis pathways on transcorneal penetration of LA-NPs/ISG in rabbit corneas. (**A**,**B**) Penetration profile (**A**) and *AUC*_0–6h_ (**B**) of Lan in LA-NPs/ISG at 4 °C and 37 °C. (**C**,**D**) Penetration profile **(C**) and *AUC*_0–6h_ (**D**) of Lan in LA-NPs/ISG using the rabbit cornea treated with endocytosis inhibitors. (**E**,**F**) Penetration profile (**E**) and *AUC*_0–6h_ (**F**) of Lan in LA-NPs/ISG using the rabbit cornea multi-treated with nystatin, dynasore, and rottlerin (Nys+Dyn+Rot). Control is the vehicle of corresponding endocytosis inhibitor. n = 5–7. * *p* < 0.05, vs. 37 °C for each category. ^#^ *p* < 0.05 vs. Control for each category. The *AUC*_0–6h_ in the LA-NPs/ISG was decreased under the 4 °C condition. In addition, the multi-treatment of nystatin, dynasore, and rottlerin also inhibited the corneal penetration of Lan in the LA-NPs/ISG.

**Figure 4:**
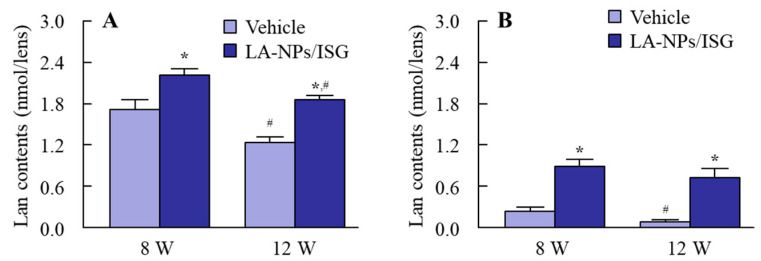
Lan contents of the lenses of SCR-N and SCR-C aged 8 and 12 weeks. (**A**,**B**) Changes in Lan content in the lenses of SCR-N (**A**) and SCR-C (**B**) repetitively instilled with LA-NPs/ISG. The repetitive instillations were performed twice per day from six weeks of age. Vehicle is solution consisting of BAC, mannitol, HPCD, and MC. n = 6. * *p* < 0.05, vs. Vehicle for each group. ^#^ *p* < 0.05, vs. 8 W for each group. The Lan content in the lenses of both SCR-N and SCR-C was increased by the repetitive instillation of LA-NPs/ISG. On the other hand, no corneal injury in the SCR repetitive instilled with LA-NPs/ISG was observed under the fluorescein stain.

Figure 3 shows the effect of energy-dependent endocytosis on the corneal penetration of LA-NPs/ISG using the isolated rabbit cornea. Although the corneal penetration of Lan was enhanced 2 h after the treatment of LA-NPs/ISG at 37 °C, the increase in Lan was prevented under the 4 °C condition, which inhibited all of the energy-dependent uptake (Figure 3A,B). Next, the changes in the corneal penetration of Lan in the rabbit cornea treated with each endocytosis inhibitor were investigated. There was no difference between the control- and cytochalasin D-treated groups (Figure 3C,D). In contrast with the results of cytochalasin D, the corneal penetration of Lan was prevented by naystatin, dynasore, and rottlerin, and *AUC*_0–6h_ in naystatin, dynasore, and rottlerin was 78.0, 86.1, and 114.4 µM∙h, respectively (Figure 3C,D). In addition, the multi-treatment of nystatin, dynasore, and rottlerin inhibited the corneal penetration more strongly than the individual inhibitors (naystatin, dynasore, and rottlerin, Figure 3E,F). Figure 4 shows the Lan transport into the lens by the instillation of LA-NPs/ISG. The Lan contents in the lenses were decreased with aging in both SCR-N and SCR-C. Moreover, the Lan content in the lenses of SCR-C was significantly lower than that in SCR-N. On the other hand, the Lan content was increased by the repetitive instillation of LA-NPs/ISG, and the Lan content of lenses in SCR-N and SCR-C repetitively instilled with LA-NPs/ISG for 6 weeks (12 weeks old SCR) were 1.5 and 6.7-fold higher in comparison with vehicle-instilled SCR, respectively.

### 3.3. Therapeutic Potential of LA-NPs/ISG on the Collapse of Lens Structure in SCR-N

**Figure 5:**
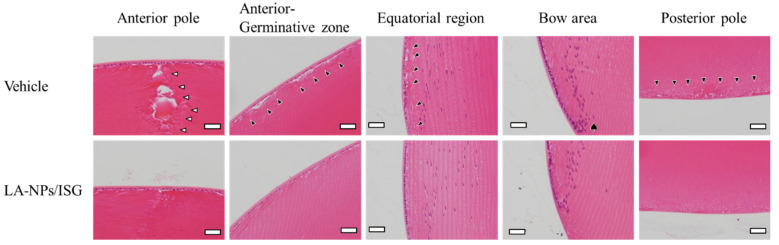
Changes in the lens structure of 12-week-old SCR-N repetitively instilled with LA-NPs/ISG. The repetitive instillation was performed twice per day from six weeks of age. White arrowhead, structure collapse of Y-shaped sutures. Black arrowhead, slight space and structure collapse. ♠, multi-layered cells. Scale bar shows 50 µm. Vehicle is solution consisting of BAC, mannitol, HPCD, and MC. The space and structure collapse in the lens of SCR-N with aging was attenuated by the instillation of LA-NPs/ISG.

Figure 5 shows the histological observation of SCR-N lenses by H.E. staining. A slight space and structure collapse (black arrowhead) were observed in the SCR-N repetitively instilled with the vehicle, and in the anterior pole, the structure collapse of Y-shaped sutures was observed due to the poor extension of lens fibers directly under the lens epithelial cell (white arrowhead). Moreover, multi-layered cells were observed just before the bow area (♠). The repetitive instillation of LA-NPs/ISG attenuated this structure collapse and the multi-layered cells in the lenses of SCR-N.

### 3.4. Delay of Lens Opacification in SCR-C by the Instillation of LA-NPs/ISG

**Figure 6:**
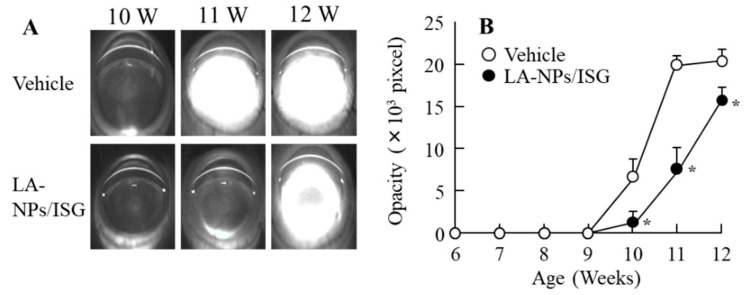
Lens opacification of Shumiya cataract rats with a combination of remarkable lens structure collapse and opacification (SCR-C) repetitively instilled with LA-NPs/ISG. (**A**,**B**) Scheimpflug slit images (**A**) and opacity levels (**B**) of SCR-C lenses repetitively instilled with LA-NPs/ISG. The repetitive instillations were performed twice per day from six weeks of age. Vehicle is solution consisting of BAC, mannitol, HPCD, and MC. n = 6. * *p* < 0.05, vs. Vehicle for each group. The onset of opacification in the SCR-C was delayed by the repetitive instillation of LA-NPs/ISG.

**Figure 7:**
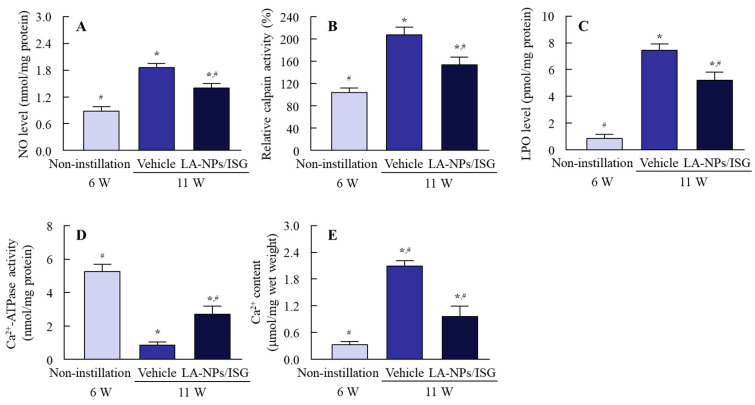
Changes in NO levels (**A**), calpain activity (**B**), lipid peroxidation (LPO) levels (**C**), Ca^2+^-ATPase activity (**D**), and Ca^2+^ content (**E**) in the lenses of 11-week-old SCR-C repetitively instilled with LA-NPs/ISG. The repetitive instillations were performed twice per day from six weeks of age. Non-injection, non-instilled SCR-C aged 6 weeks. Vehicle, Vehicle-instilled SCR-C aged 11 weeks. LA-NPs/ISG, LA-NPs/ISG instilled SCR-C aged 11 weeks. Vehicle is solution consisting of BAC, mannitol, HPCD, and MC. n = 6–13. * *p* < 0.05, vs. Non-instillation for each group. ^#^ *p* < 0.05, vs. vehicle for each group. The repetitive instillation of LA-NPs/ISG attenuated the changes in cataract-related factors (enhancement of NO levels, calpain activity, LPO levels, and Ca^2+^ content, and decrease of Ca^2+^-ATPase activity) in the lenses of SCR-C.

Figure 6 shows the delay of lens opacification in SCR-C by the repetitive instillation of LA-NPs/ISG. The lens opacification in SCR-C repetitively instilled with the vehicle began at nine weeks of age, and the mature cataracts had formed at 11 weeks. Repetitive instillation of LA-NPs/ISGs significantly retarded the progress of lens opacification, although the repetitive instillation could not reverse or stop lens opacification. Figure 7 shows the preventive effect of LA-NPs/ISG on the NO levels, calpain activity, LPO levels, Ca^2+^-ATPase activity, and Ca^2+^ content in the lenses of SCR-C. It is known that these NO levels, calpain activity, LPO levels, Ca^2+^-ATPase activity, and Ca^2+^ content in the lenses are changed in the progress of lens opacification in SCR-C, and the inhibition of these factors leads to a delay in the onset of cataract development [31]. In the 11-week-old SCR-C with opaque lenses, the NO levels, calpain activity, LPO levels, and Ca^2+^ content were significantly increased, and the Ca^2+^-ATPase activity was decreased, in comparison with 6-week-old SCR-C with transparent lens. The repetitive instillation of LA-NPs/ISG attenuated the changes in these cataract-related factors (NO, calpain activity, LPO, Ca^2+^-ATPase activity, Ca^2+^ content) in the lenses of SCR-C.

## 4. Discussion

In the delivery of the Lan into lenses after instillation, the prolongation of preocular residence and enhancement of corneal permeability are important. We previously prepared an in situ gelling system based on MC and tranilast nanoparticles, and we showed that the instillation of in situ gel containing tranilast nanoparticles provided high absorption and the prolongation of preocular residence of tranilast [17]. In the present study, we attempted to design ophthalmic in situ gel formulations containing Lan nanoparticles (LA-NPs/ISG) and measured the characteristics, delivery pathway into the lens, and anti-cataract effects of LA-NPs/ISG.

In situ gels are viscous liquids that, upon exposure to physiological conditions, shift to a gel phase, leading to an increase of ocular residence time [32,33]. Thermosensitive in situ gels, which undergo a sol–gel transition upon heating or cooling because of changes in the intermolecular interaction, are more promising for sustained ocular drug delivery. The MC component is water-soluble non-ionic cellulose ether, and inverse thermal gelling forms a physically cross-linked hydrogel at physiological temperatures. MC is frequently used as a gelling agent [34]. In this study, MC was selected as the base of the in situ gel and 0.005% BAC, 0.5% mannitol, and 5% HPCD were used as other additives to prepare the in situ gel containing Lan nanoparticles. BAC (0.005%) is a quaternary ammonium compound, and it is used as a preservative in eye drops. It is known that BAC significantly alters precorneal mucins [35] or induces oxidative stress [36,37], and causes corneal toxicity, and that 0.5% mannitol prevents this BAC toxicity [38]. Therefore, mannitol can be added to attenuate the corneal toxicity caused by BAC. Moreover, it has been reported that MC enhances the crushing efficiency in the bead mill treatment, and the bead mill treatment with MC can provide drug particle sizes of approximately 60–200 nm [23,39]. Furthermore, the addition of HPCD prevents aggregation and enhances the stability of nano-dispersions [23]. Moreover, HPCD at levels less than 12.5% does not result in observable irritation of the eye membrane [40]. In light of these previous findings, we prepared ophthalmic formulations containing Lan nanoparticles and achieved particles sized 60–250 nm. In addition, the viscosity of LA-NPs/ISG was enhanced under the 37 °C condition.

Furthermore, we demonstrated the corneal toxicity by the instillation of LA-NPs/ISG. In the instillation of ophthalmic formulations, drugs are diluted to approximately 20% by lacrimal fluids. Moreover, drugs on the ocular surface are excreted though the nasolacrimal duct 2 min after instillation [41]. Therefore, we selected a stimulation time of 2 min and determined cell toxicity using HCE-T cells (Figure 2A). The vehicle decreased the cell viability in comparison with saline. BAC (0.005%) was included in the vehicle as a preservative. Although it is known that corneal stimulation is a side effect of BAC, the cell viability in treatment with the vehicle was lower than that in cells treated with 0.005% BAC only (61.1 ± 4.9%, n = 7). These low stimulations in the vehicle containing 0.005% BAC were due to the presence of mannitol, since we previously found that mannitol prevents corneal stimulation by BAC [38]. The viability in HCE-T cells treated with LA-NPs/ISG was similar to those treated by the vehicle. Moreover, it is important to evaluate the effect of LA-NPs/ISG on corneal wound healing in in vivo experiments. Therefore, we also investigated the in vivo corneal toxicity using rats’ debrided corneal epithelia (Figure 2B,C). The repetitive instillation did not cause corneal damage in the rabbits. In addition, corneal wound healing showed no difference between rats instilled with saline or with LA-NPs/ISG. Moreover, in the study of Figure 4, no corneal injury in the SCR repetitive instilled with LA-NPs/ISG was observed under the fluorescein stain. These results showed that the repetitive instillation of LA-NPs/ISG was able to be applied as a therapy for eye disease.

Next, we demonstrated whether the instillation of LA-NPs/ISG delivered the Lan into the lens. In order for the drug after instillation to reach the lens, it needs to pass through the cornea and shift to the intraocular side. Therefore, the corneal penetration of LA-NPs/ISG was measured in this study. The Lan in LA-NPs/ISG penetrated the cornea (Figure 3A), and the Lan content in the lenses of SCR was enhanced by the repetitive instillation of LA-NPs/ISG (Figure 4). In addition, the Lan content in the lens instilled with Lan nanoparticles containing 1.5% MC was higher than that in Lan nanoparticles containing 0.5% MC (SCR-N 1.67 ± 0.20, SCR-C, 0.44 ± 0.05, nmol/lens, n = 5). Moreover, we examined the mechanism of corneal penetration after the instillation of LA-NPs/ISG. In previous research, it was reported that energy-dependent endocytosis, such as CavME [25], CME [26], MP [27], and phagocytosis [25], was related to the take-up into the corneal epithelium, resulting in increasing rates of transcorneal penetration of nanoparticles [16]. In the LA-NPs/ISG, the energy-dependent endocytosis enhanced the corneal penetration, since the *AUC*_0–6h_ in the LA-NPs/ISG was significantly decreased under the 4 °C condition (Figure 3A). Moreover, we investigated whether the corneal penetration was inhibited by each inhibitor of energy-dependent endocytosis and found that the multi-treatment of nystatin, dynasore, and rottlerin attenuated the corneal penetration of Lan in the LA-NPs/ISG (Figure 3E,F). These results showed that LA-NPs/ISG were taken into the cornea by CavME, CME, and MP, and then released to the intraocular side. Then, the Lan in the aqueous humor shifted to the lens, and the Lan content in the lens was enhanced.

SCR-N, with a slight collapse of lens structure (not opacification), was a suitable model to evaluate the therapeutic effect of drugs on early cataract development. A slight space and structure collapse in the anterior-germinative zone and equatorial region, poor extension of lens fibers directly under the lens epithelial cell, and multi-layered cells before the bow were observed (Figure 5). The space and structure collapse in the lens of SCR-N was prevented by the repetitive instillation of LA-NPs/ISG (Figure 5). The SCR-C carries a specific combination of hypomorphic mutations of Lan synthase and FDFT1 (farnesyl diphosphate farnesyl transferase 1) genes, and the Lan levels were lower than that in normal and SCR-N [20]. The mutation of the Lan synthase gene results in decreased Lan and cholesterol levels in the lens, causing lens opacification [20]. Furthermore, the anti-cataract effect for serious cataract development was also investigated using the SCR-C with lens opacification. The instillation of LA-NPs/ISG delayed the onset of opacification in the SCR-C, although repetitive instillation could not reverse or stop lens opacification. The reversal of opacity by Lan has been the subject of some discussion. Researchers have shown that Lan treatment can improve crystallin aggregation and restore transparency to opaque lenses [6]. In contrast, other studies have failed to show binding to aggregated lens proteins to dissolve cataracts [42]. This discrepancy may be caused by the difference in the period and dose of Lan supplement or by differences in the models used. In this study, the difference in the Lan contents of lenses of SCR with and without LA-NPs/ISG were approximately 0.6 nmol/lens (Figure 4), and the Lan content in the lenses of SCR-C repetitively instilled with LA-NPs/ISG for six weeks was 6.7-fold higher in comparison with that in saline-instilled SCR-C. However, the Lan content in lenses of SCR-C repetitively instilled with LA-NPs/ISG was lower than that in corresponding SCR-N (Figure 4). A greater supplement of Lan content may be needed to reverse crystallin aggregation and restore transparency to opaque lens. Moreover, we previously reported that the serious structural collapses with posterior movement of the lens nucleus were observed in the SCR-C [24], and the serious structural collapses was not reversed by the instillation of LA-NPs/ISG in this study. Further studies are needed to investigate whether the treatment of Lan can restore opaque lenses to transparency.

In addition to differences in the period and dose of the Lan supplement, it has been suggested that differences in factors related to cataract onset in animal models are also involved in the anti-cataract effect of Lan. Previous reports showed that NO via iNOS decreased Ca^2+^-ATPase activity by lipid peroxidation and caused Ca^2+^ dysfunction in the lenses of SCR-C. Enhanced Ca^2+^ by Ca^2+^ dysfunction increased the degradation of lens proteins via calpain activity, resulting in lens opacification [31]. Furthermore, we previously reported that the space and structure collapse in lenses with Lan deficiency caused excessive iNOS production [24]. In this study, the repetitive instillation of LA-NPs/ISG decreased the changes in these cataract-related factors (NO, calpain activity, LPO, Ca^2+^-ATPase activity, Ca^2+^ content) in the lenses of SCR-C (Figure 7). These results suggest that the repetitive instillation of LA-NPs/ISG delivered the Lan into the lenses and prevented the slight collapse of the lens structure of SCR-N (Figure 5). However, Lan supply by the repetitive instillation of LA-NPs/ISG was not sufficient to restore the Lan content in the lenses of SCR-C with marked Lan deficiency. Further studies are needed to design anti-cataract drugs to stop the progression of lens opacification and restore transparency to opaque lenses. In future work, we plan to measure the therapeutic effect of in situ gelling formulations based on Lan nanoparticles/iNOS inhibitor in SCR-C.

## 5. Conclusions

We developed ophthalmic in situ gel containing Lan nanoparticles and found that the repetitive instillation of LA-NPs/ISG delivered the Lan into the lenses and prevented the slight collapse of the lens structure of SCR-N. Moreover, the LA-NPs/ISG retarded the onset of lens opacification in SCR-C.
